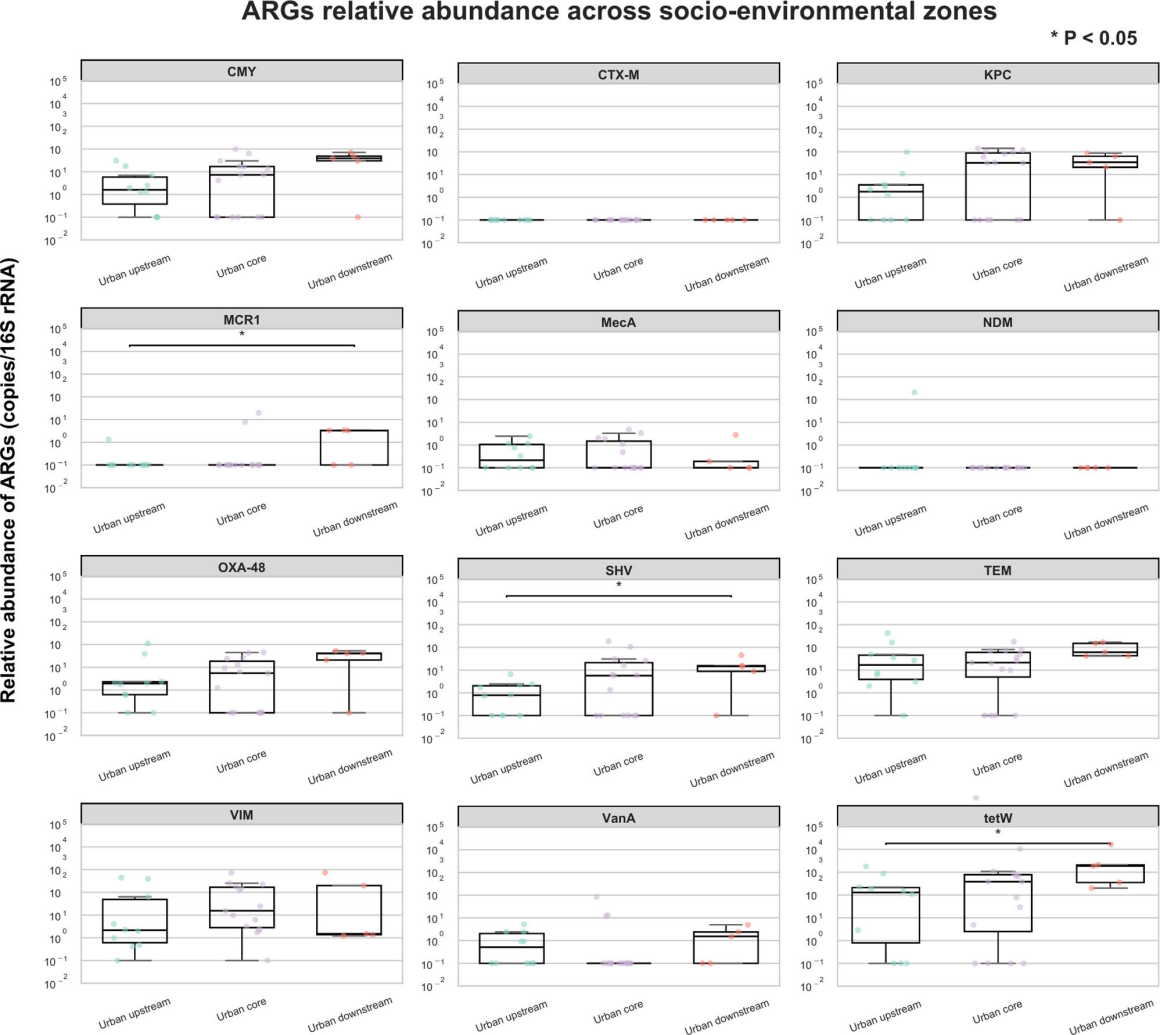# 56 The Prevalence of Uropathogens and Antimicrobial Susceptibility Profiles Encountered in Western India.

**DOI:** 10.1017/ash.2026.10489

**Published:** 2026-06-23

**Authors:** Erika Paola Viana Cardenas

**Affiliations:** 1 Stanford University

## Abstract

**Background:** Antimicrobial resistance (AMR) is a growing global health threat, yet surveillance systems remain heavily centered on clinical reporting and often overlook environmental transmission pathways. Urban rivers receiving mixed municipal, hospital, and agricultural inputs may act as important reservoirs and dissemination routes for antibiotic resistance genes (ARGs), particularly in resource-limited settings. Understanding spatial patterns of environmental resistomes is essential for advancing One Health–informed AMR surveillance. **Methods:** We conducted a longitudinal, multi-site investigation of ARG occurrence along the Cuautla River, an urban watershed in Morelos, Mexico, serving the resource-limited city of Cuautla. Surface water samples were collected across six locations spanning urban upstream (L1-2), urban core (L3-5), and urban downstream zones (L6) (Figure 1). Targeted droplet digital PCR (ddPCR) was used to quantify relevant ARGs, including Gram-positives (mecA and vanA), Gram-negatives beta-lactamases (CTX-M and SHV), AmpC-type β-lactamase (CMY), and Carbapenemase (KPC, NDM, VIM, IMP, and OXA-48), tetracycline (tetW) and Polymyxin-resistant (MCR-1). Physicochemical parameters and fecal indicator bacteria were measured concurrently to contextualize microbial contamination. Spatial and temporal trends in ARG abundance and composition were evaluated using non-parametric statistical analyses. **Results:** ARGs were widely detected across the watershed, with particularly high abundances of tetW, CMY, TEM, and multiple Gram-negative carbapenemase genes, including KPC, OXA-48, and VIM. The plasmid-mediated colistin resistance gene mcr-1 was also detected at multiple sites, highlighting the environmental presence of resistance determinants associated with last-resort antimicrobials (Figure 2). ARG abundances generally increased from urban upstream to urban downstream locations, likely reflecting cumulative inputs from domestic wastewater, healthcare facilities, and densely populated areas. Although ARG loads peaked within the urban core, elevated resistance signals remained detectable in downstream urban edge regions (Figure 3). In contrast to largely compliant physicochemical conditions, fecal indicator bacteria revealed pervasive microbial contamination across urban sites. **Conclusions:** Our findings demonstrate extensive resistome contamination within an urban river system, encompassing both environmental and clinically important ARGs. The spatial accumulation of resistance signals downstream and the detection of colistin resistance genes underscore the role of river ecosystem as conduits linking human activity to broader environmental AMR dissemination. These results highlight the need to integrate environmental water surveillance into AMR monitoring frameworks and support a One Health approach that connects wastewater management, antibiotic stewardship, and public-health protection in resource-limited settings.

**Figure:**
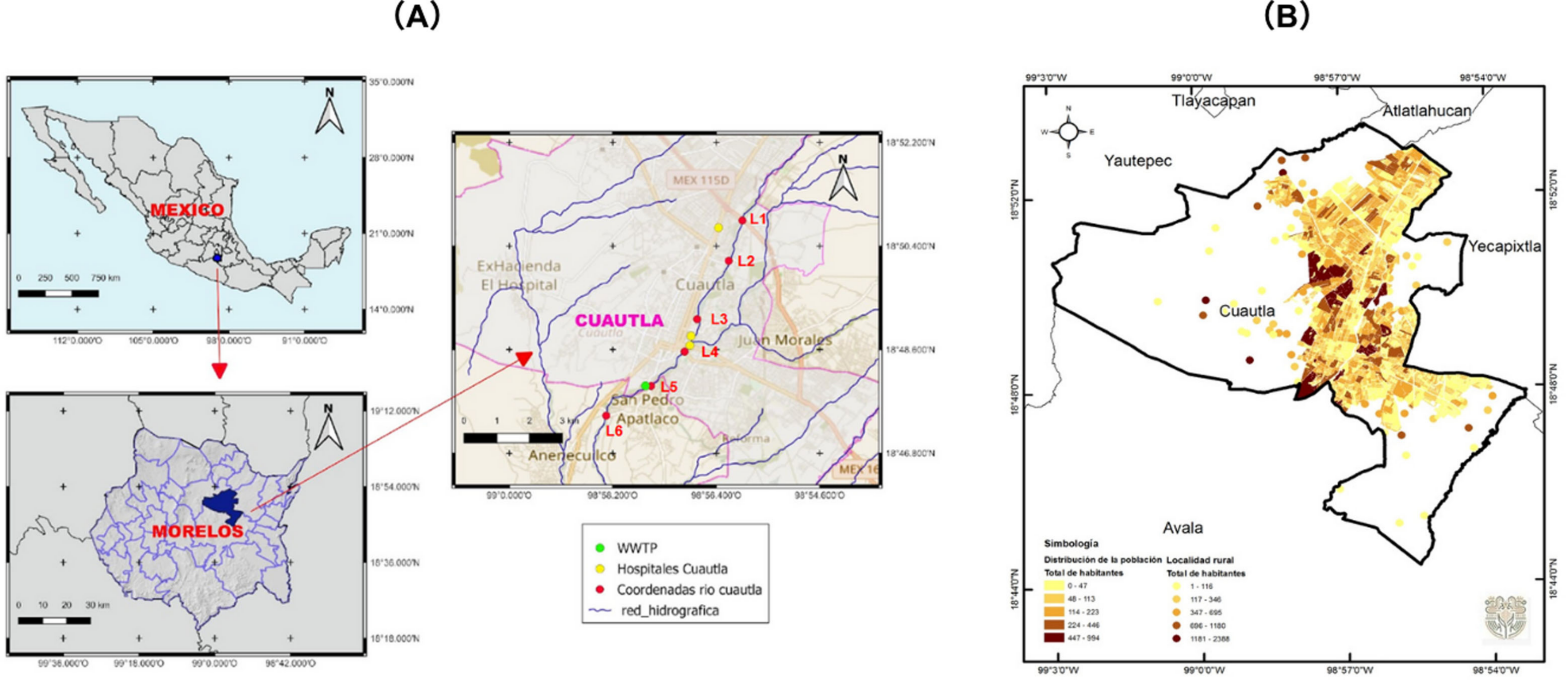

**Figure:**
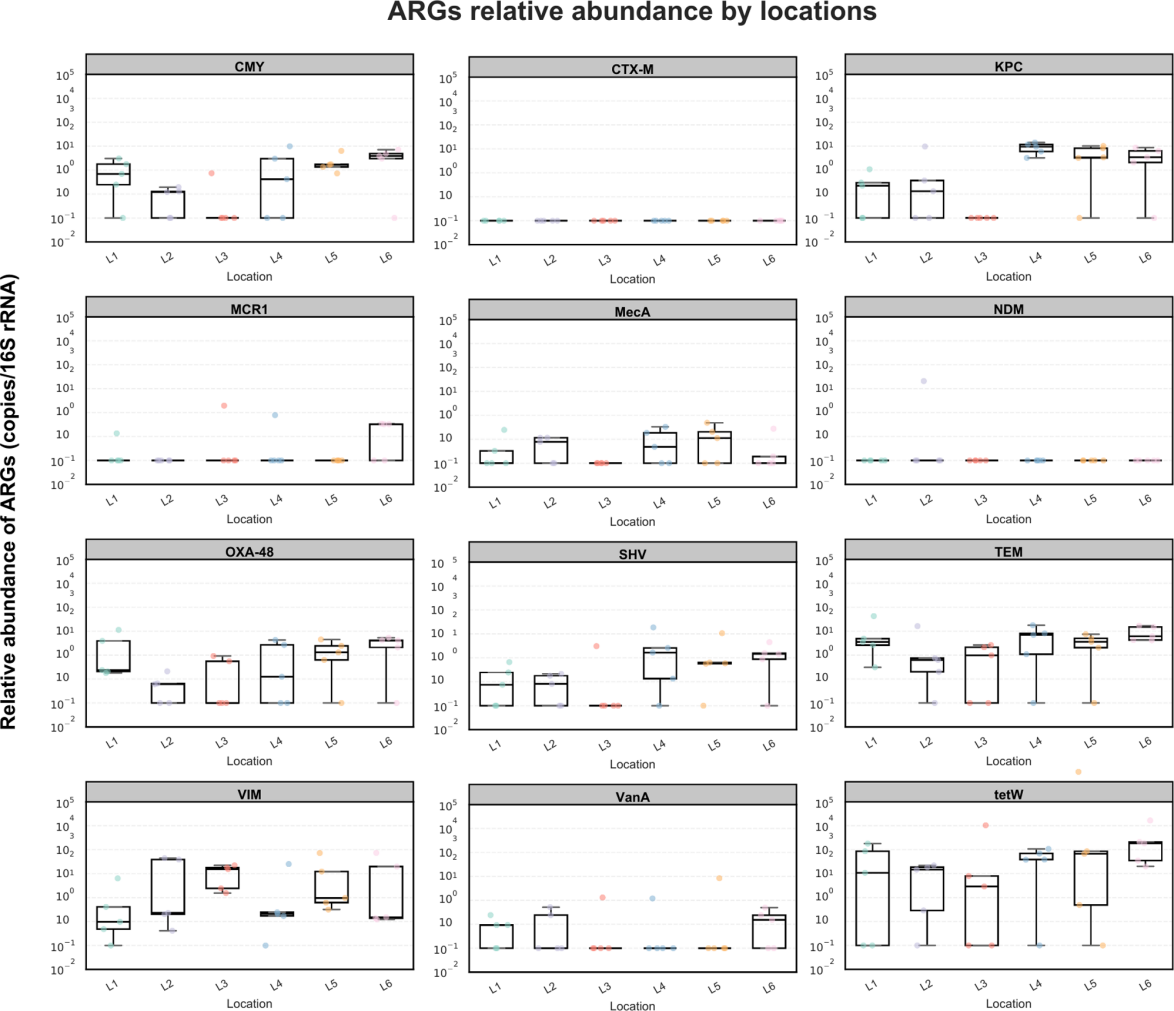

**Figure:**